# The Density of Teeth as Influenced by the Food and by the Administration of Lime-Salts

**Published:** 1886-11

**Authors:** W. D. Miller

**Affiliations:** Berlin


### Independent Practitioner.

VOL. VII. NOVEMBER, 1886. NO. 11.

### Original Communications.

NOTE.—No paper published or to be published in another journal will be accepted for this department. All papers must be in the hands of the Editor before the first day of the month preceding that in which they are expected to appear. Extra copies will be furnished to each contributor of an accepted original article, and reprints, in pamphlet form, may be had at the cost of the paper, press-work and binding, if ordered when the manuscript is forwarded. The Editor and Publishers are not responsible for the opinions expressed by contributors. The journal is issued promptly, on the first day of each month.

### THE DENSITY OF TEETH AS INFLUENCED BY THE FOOD AND BY THE ADMINISTRATION OF LIME-SALTS.

BY DR. W. D. MILLER, BERLIN.

ABSTRACT OF A LECTURE DELIVERED BEFORE THE AMERICAN DENTAL SOCIETY OF EUROPE, AUGUST 2D, 1886.

While many elaborate experiments have been made by different physiologists of celebrity for the purpose of determining the action of inorganic substances, particularly phosphate of lime, upon the osseous system, either fully developed or developing, no experiments of like nature have been made upon the teeth. We have relied altogether upon clinical evidence, which has been so conflicting that, notwithstanding the fact that this question has never ceased to be discussed for the last fifty years, we are not much nearer the solution. It is utterly impossible for anyone to eliminate all the sources of error in observations upon human beings. No one can bring two individuals under exactly the same conditions, with the exception that one receives lime-salts and the other none, nor can the proper value be assigned to the many other factors which at the same time are in operation. Consequently, different observers secure results entirely different, and often the same observer finds lime-salts in one case apparently producing most beneficial results, and in another none at all. Those who have administered lime-salts to a gravid woman, and have seen the child grow up with strong teeth, have attributed this to the lime-salts; while those who, under like circumstances have found poor teeth, have accused the lime-salts. In both cases the result may have been brought about by entirely different factors.

But this phase of the question has been so thoroughly presented by Dr. Barrett (INDEPENDENT PRACTITIONER, vol. vi, page 453), that it does not require further discussion here.

This subject may, I think, be best studied under four heads.

1. Can it happen that a person who daily takes a normal quantity of food may receive less lime-salts than are required to build up the osseous and dental systems?

2. What effects have been produced upon the osseous system of animals by varying the amount of lime-salts taken in?

3. What effect upon the composition of the teeth of adult animals?

4. What effect upon the development and composition of the teeth of growing animals?

I must state in the beginning, by way of apology, that the work which I have undertaken is by no means complete, yet I present this paper as an introduction to the subject now, since the experiments yet to be made will require eight or nine months for their completion.

The following table which I have worked out, principally by the help of *Koenig's die Mënslichen Genussund Nahrœngs–Mittel*, will very materially aid us in the attempt to answer the first question. In this table the figures indicate the amount of each constituent in 100 parts of substance analyzed. The numbers for bread (knowing the amount of saline matter present) were calculated on the supposition that the different constituents maintain the same proportions in bread as in flour:

| | Water. | Ash. | Potassium. | Sodium. | Lime. (CaO) | Magnesium. | Phosphoric Acid. ($PO_4$) |
|---|---|---|---|---|---|---|---|
| Meat of ruminants and fowls. | 62.00 | 1.9 | 0.75 | 0.20 | 0.049 | 0.064 | 0.828 |
| Beef extract | ...... | ...... | 7.35 | 2.27 | 0.08 | 0.55 | 5.25 |
| Eggs | 74.00 | ...... | 0.156 | 0.208 | 0.098 | 0.1014 | 0.34 |
| Wheat | 13.65 | ...... | 0.56 | 0.032 | 0.051 | 0.22 | 0.91 |
| Fine wheat flour | 13.34 | 0.48 | 0.14 | 0.003 | 0.031 | 0.034 | 0.21 |
| Coarse wheat flour | 12.65 | 0.96 | 0.216 | 0.0066 | 0.044 | 0.077 | 0.35 |
| Fine wheat bread | 35.59 | ...... | 0.37 | ..... | 0.082 | ...... | 0.54 |
| Coarse wheat bread | 40.45 | ...... | 0.38 | ...... | 0.072 | ...... | 0.61 |
| Rice (bolted) | 13.11 | 1.01 | 0.195 | 0.046 | 0.028 | 0.095 | 0.462 |
| Rice (unbolted) | 9.55 | 4.90 | ..... | ...... | ...... | ...... | ..... |
| Beans | 14.76 | 3.26 | 1.28 | 0.032 | 0.15 | 0.21 | 0.18 |
| Potatoes | 75.00 | ...... | 0.56 | 0.027 | 0.0226 | 0.045 | 0.16 |
| Turnips | 86.00 | ...... | 0.40 | 0.07 | 0.126 | 0.064 | 0.25 |
| Cauliflower | 91.00 | ...... | 0.267 | 0.103 | 0.189 | ..... | 0.132 |
| Cabbage | 87.00 | ..... | 0.34 | ...... | 0.62 | ...... | 0.06 |
| Peas (bolted) | 12.73 | 1.75 | ..... | ...... | ...... | ...... | ...... |
| Codfish | 81.00 | ...... | 0.29 | 0.76 | 0.072 | 0.04 | 0.29 |
| Bean flour | 10.84 | 2.95 | ...... | ...... | ...... | .... | ...... |
| Oatmeal | 10.07 | 2.24 | ..... | ...... | ...... | ...... | ...... |
| Sago meal | 16.14 | 0.22 | ...... | ...... | ...... | ...... | ...... |
| Tapioca | 14.43 | 0.25 | ...... | ...... | ...... | ...... | ...... |
| Ox (heart) | 70.08 | 0.78 | ...... | ...... | ...... | ...... | ...... |
| Ox (lung) | 81.03 | 3.39 | ...... | ...... | ...... | ...... | ...... |
| Woman's milk | 87.02 | 0.45 | ...... | ...... | 0.075 | ...... | 0.102 |
| Cow's milk | ...... | 0.71 | ...... | ...... | 0.164 | ...... | 0.198 |
| Hog's milk | 84.04 | 1.05 | ...... | ...... | ...... | ...... | ...... |
| Beer (porter) | 87.60 | 0.419 | ...... | ...... | 0.008 | ...... | 0.092 |
| Water (Berlin) | ...... | ...... | ...... | ...... | 0.0141 to 0.0612 | ...... | ...... |

If now we suppose an individual to consume on an average daily 1 lb. bread, 1 lb. meat, 1 lb. potatoes, 2¼ lbs. beer and 2 lbs. water, we may make the following calculation:

| | CaO. | $PO_4$. |
|---|---|---|
| 1 lb. meat contains | 0.000491 | 0.00828 |
| 1 " bread " | 0.000820 | 0.00540 |
| 1 " potatoes " | 0.000226 | 0.00160 |
| 2¼ " beer " | 0.000180 | 0.00213 |
| 2 " well-water contains | 0.000753 | ——— |
| Total in one day | 0.002470 | 0.01741 |
| " " " year | 0.90155 | 6.35465 |

A young man gives out through the urine yearly 0.095 CaO and 0.15 $PO_4$. Subtracting these amounts, we have the total amount taken and retained in the system yearly, 0.8065 CaO and 6.20 $PO_4$. This, of course, would be reduced by the amount which passes out with the fæces, which may be very little or very much, depending upon the condition of the digestive and absorptive apparatus. Leaving this out of consideration for the present, we easily determine how many years it will require for one to take in enough lime-salts to build up his osseous system. A skeleton weighing 24 lbs. has, according to Heinby's analysis, 9.26 lbs. lime and 12.9 phosphoric acid, and at the rates of 0.80655 lbs. CaO and 6.20 lbs. $PO_4$ yearly, it would take 12.4 years and 2.1 years respectively to acquire the necessary amount of lime and phosphoric acid.

From this calculation it will be seen that while we, under the specified regime, have an abundance of phosphoric acid, we have by no means an excess of lime. I have endeavored throughout to take average numbers, and while many will take in more lime-salts, either by consuming more food or food richer in lime (beans, cabbage, oats, milk,* water in lime districts), others without doubt receive less salts, either from being unable to procure the necessary amount of food, or living too exclusively on foods which are poor in lime-salts. Children fed on sago, tapioca, etc., receive, beyond doubt, less lime than is necessary to properly build up the osseous system.

A woman lactating at the rate of 1 liter per day, will give out 0.616 lbs. in a year, besides the loss by excretion. This is more than would be taken in under a regime poor in lime, and would entail a withdrawal of lime-salts from the system, as shown by the experients of Forster, the soft tissues being affected first, then the bones, and last, if at all, the teeth. For this evil the remedy is evident; a sufficient quantity of plain, wholesome food, not notoriously poor in lime.

Dr. Casse, director of a hospital for rachitic children in Middelkirke, near Brussels, has constantly between one and two hundred rachitic children under his charge. He informs me that he uses no other remedies than good air and wholesome food, milk, peas,

---

*A quart of cow's milk daily will alone furnish in eight years as much lime as is contained in an ordinary skeleton.

beans, etc., forming a prominent factor in the regular diet of the children. Under this regime marvellous cures are accomplished. Kirk saw a marked improvement in the teeth of the children of a Philadelphia hospital, resulting from a diet rich in lime.

Another very important question is suggested by these facts. Supposing that the food contains less lime than is needed by the patient, can we make good the deficiency by administering the phosphate of lime as a medicament? This brings us to the question given under the second heading—What effects have been made upon the osseous system of animals by varying the amount of lime-salts taken in?

The combined results of a very great number of experiments indicate that in the adult animal changes in the osseous system take place very slowly, and Hoppe-Seyler asserts that it is impossible to effect any change in the chemical composition of the bones by withholding the normal amount of lime-salts, or by administering an excess. The experiments of Foerster (given below) hardly bear out the statement of Hoppe-Seyler. That in the case of young animals the administration of lime-salts, where they are not present in normal quantities in the food, will result in great good, is clearly proved by the experiments of Voit, Tuczek, Hofmeister, and many others. Voit and Tuczek fed young doves on wheat which had been freed from lime and phosphoric acid. They soon became ill, and died with symptoms characteristic of rickets, whereas doves treated in the same manner, with the addition of small pieces of mortar, developed normally. Young dogs fed with a mixture of meat and bacon, containing very little lime phosphate, exhibited the same symptoms, which were completely relieved by administering bone-ash. The skeleton of the dogs in the first series contained 71.9 per cent. water, in the second only 64.9 per cent.

Here the lack of a sufficient quantity of salts in the food was made good by administering the mineral. I am, moreover, at a loss to see what difference it can make, as far as the capacity for resorption and assimilation is concerned, whether we administer a grain of phosphate of lime in the form of a beefsteak, or as bone-ash, or as chemically pure phosphate of lime. After it has been subjected to the hydrochloric acid of the stomach, it will in both cases be reduced to identically the same form. In both cases we obtain a solution of the phosphate in hydrochloric acid, then a decomposition of the phosphate, or a part of it, setting free phosphoric acid and forming chloride of lime. A part may remain undecomposed, as phosphate of lime, particularly if the gastric juice is deficient in hydrochloric acid.

I should say, then, that, especially in the case of young animals, the development of the osseous system may be markedly influenced by the quantity of salts in the food, and that in case the foods contain too small a quantity, it may be made good by administering the necessary amount. The first requisite for the development of a good osseous system is the proper quantity of lime and phosphoric acid, and to secure this, it is not altogether immaterial what class of food is taken, as the above table will show. If a man could live on tapioca pudding alone, and consume two pounds of the starch a day, it would require about 150 years for him to take up enough lime to make his bones, to say nothing of the rest of his body, taking no account of the waste by excretion.

The second requisite is the absorption, and the third the assimilation of the lime and phosphoric acid. If either of the last two requisites are not fulfilled, the administration of lime-salts will probably not accomplish the desired end. It would not do to say it will not accomplish anything, since even in cases of defective absorption and assimilation, the amount of salts which is utilized may bear a certain proportion to the amount presented.

In answer to the second question, we would say, then, that the fully formed bones of the adult animal respond very slowly to any change in the normal amount of lime taken with the food, and that even in lime-hunger the bones do not readily give up their phosphates (Weiske, Zalesky, Hoppe-Seyler, etc.), but that the bones of young animals are very sensible to any restriction in the requisite quantity of saline matters, and that any lack of lime in the food may be made good by administering it in form of bone-ash, or lime-salts and phosphates.

We come now to the third question. Can the chemical composition of adult *teeth* be changed by a change in the quantity of lime-salts in ordinary food? In other words, can the lime-salts of the teeth be withdrawn for use elsewhere, or excreted?

The fact that some bone gives up its lime-salts very tardily under all circumstances, would lead us to infer that the same would be the case with the teeth, even in a greater degree. On the other hand, the rapid breaking down of the teeth during pregnancy has been almost universally attributed to the robbing of the teeth of the mother of their lime-salts, to build up the fœtus. While dentists urge a diet very rich in lime, others restrict the pregnant woman to a diet containing but little lime, in order that the bones of the child may not become so rigid as to increase the difficulties of childbirth.

These ideas may all be defended. A number of experiments of Forster (Zeitschr. f. Biol. Bd. IX, S. 297) show that under continued feeding with ash-free substances, the animals not only suffer from inanition, but that under these conditions more lime is excreted than can be taken from the soft tissues, consequently a part of it must come from the bones.

This results, according to Zalesky and Hoppe-Seyler, not in a change in the chemical composition of the bones, but simply in rarefaction, the organic and inorganic parts being removed simultaneously, as in resorption.

A case in which a change in the chemical composition of the teeth seems to have taken place, recently came under my notice. For three or four years I have had the care of the teeth of a well-known pathologist in Berlin. They are of exceeding poor structure, very soft, and seem to offer scarcely any resistance whatever to the agents which produce decay. When I called the attention of the patient to this fact, he told me that a few years ago he did not have a trace of decay about his teeth, and that he had always been told by dentists that his teeth were of the best structure and could never decay. He also stated that for some years past his urine has constantly contained "enormous quantities" of lime-salts. He had recently detected certain defects in his osseous system, which he had thought accounted for the excess of lime in his urine. He now wished to know whether a part of this excess did not come from the teeth, thereby accounting for their diminished hardness. But many cases of this kind, where hard teeth have apparently become very soft and soft teeth very hard, are no doubt familiar to every practitioner, and do not help much toward a solution of the question.

We have seen that while authorities agree as to the effect produced upon young animals by withholding the normal amount of lime-salts, they differ very widely as to the effect of lime hunger upon adult bones, nor are the views of dentists regarding the possibility of a change in adult dentine any less at variance, though I am not aware that any experiments upon lower animals, the only means by which the question can at present be solved, have yet been made.

The experiments which I have undertaken upon this point were begun upon three grown and two young dogs. Unfortunately, one of the young dogs died from the anæsthetic, and the other got a violent diarrhœa, as a result of being deprived of the necessary inorganic materials, of which he died before the experiment progressed far enough to obtain any results. From each of the three grown dogs I extracted, under anæsthesia, with great difficulty, an eye-tooth. The dogs were then put on a diet of fresh bacon and sugar, which they took very well for about two weeks, when they refused to take their food, but by slightly frying the bacon and occasionally adding small pieces of mashed meat (the salts being mostly removed), I succeeded in keeping them on their legs. After three months another tooth was taken from each dog, and the food changed to horse-meat, with salts of lime. A very marked change was brought about in their condition in a short time. After fifteen weeks, *i. e.*, about seven months from the beginning of the experiments, they were brought to a close, and the dentine of the teeth analyzed, showing the following proportion of lime-salts:

| | First. | Second. | Third. | Average. |
|---|---|---|---|---|
| At beginning of experiment........... | 72.66 | 72.35 | 70.77 | 71.93 |
| After 3 months....................... | 71.52 | 72.25 | 70.83 | 71.80 |
| After 15 weeks..................... | 73.71 | 71.70 | 71.86 | 72.22 |

From the first of the experiments it appears as though a marked change had been produced, a falling off of 0.86 per cent. during the first twelve weeks, and a rise of 1.59 per cent. during the last fifteen weeks. This result, however, is not borne out by the other experiments. The second shows a slight fall during the first period, but no rise during the second, while the third shows a rise during the second period, but no fall during the first. If we take the average of the three results we get a fall of 0.13 per cent. during the first, and a rise of 0.42 per cent. during the second period. Although, on the whole, it might appear that a change had been produced in the proportion of lime-salts to organic matter in the teeth, yet the number of experiments is too small and the results too little pronounced to admit of drawing any definite conclusions.

As simple as these experiments appear, they are not without a number of difficulties. The length of time necessary, the difficulty of securing lime-free food which the animals will take, the many sources of error in the analyses by which slight changes may escape detection, the very considerable expense accompanying the experiments ($30 to $40 per dog), are some of them. Nevertheless, I hope in twelve months more to obtain results which will throw some light upon the third and fourth questions suggested at the beginning.

---

In the discussion which followed, Dr. Jenkins called attention to one source of elimination of lime-salts not mentioned, viz., salivary calculus. He was also of the opinion that the acid condition of the buccal fluids was a prolific source of caries during pregnancy.

Dr. Miller replied, thanking Dr. Jenkins for the mention of salivary calculus, which, in many cases, is a source of loss not to be overlooked. As for the caries of pregnancy, he had not attempted to furnish an explanation for that. His object had been to show that, under certain conditions, the system, particularly of the child, less frequently of the adult, does not receive enough lime to meet the requirements, and that in such cases attention should be given to the character of food taken, and in case it is poor in lime-salts they may with advantage be administered as a medicament.

